# Ultra-Compact 100 × 100 μm^2^ Footprint Hybrid Device with Spin-Valve Nanosensors

**DOI:** 10.3390/s151229809

**Published:** 2015-12-04

**Authors:** Diana C. Leitao, Paulo Coelho, Jerome Borme, Simon Knudde, Susana Cardoso, Paulo P. Freitas

**Affiliations:** 1 INESC-MN - Instituto de Engenharia de Sistemas e Computadores - Microsistemas e Nanotecnologias and IN - Institute of Nanoscience and Nanotechnology, Rua Alves Redol 9, Lisboa 1000-029, Portugal; paulo.coelho18@gmail.com (P.C.); sknudde@inesc-mn.pt (S.K.); scardoso@inesc-mn.pt (S.C.); pfreitas@inesc-mn.pt (P.P.F.); 2 Instituto Superior Técnico, Universidade de Lisboa, Av. Rovisco Pais, Lisboa 1000, Portugal; 3 INL - International Iberian Nanotechnology Laboratory, Av. Mestre José Veiga, 4715 Braga, Portugal; jerome.borme@inl.int

**Keywords:** spin valves, nanosensors, flux guides

## Abstract

Magnetic field mapping with micrometric spatial resolution and high sensitivity is a challenging application, and the technological solutions are usually based on large area devices integrating discrete magnetic flux guide elements. In this work we demonstrate a high performance hybrid device with improved field sensitivity levels and small footprint, consisting of a ultra-compact 2D design where nanometric spin valve sensors are inserted within the gap of thin-film magnetic flux concentrators. Pole-sensor distances down to 400 nm are demonstrated using nanofabrication techniques combined with an optimized liftoff process. These 100 × 100 μm${}^{2}$ pixel sensors can be integrated in modular devices for surface mapping without moving parts.

## 1. Introduction

Recent developments in magnetic field imaging techniques have resorted to the use of magnetoresistive (MR) sensors for magnetic scanning probe microscopy [1,2]. Profiting from the maturity of MR read-heads for magnetic recording, spin-valve (SV) sensors with sub-μm lateral dimensions and enhanced sensitivities [3,4] become excellent candidates for high-speed force microscopy [5]. The latter requires highly sensitive and highly spatially-resolved sensors, conditions that can be straightforwardly met by SVs.

While the spatial resolution is mostly dependent on the size of the structures, the field sensitivity of SV sensors can be improved by incorporating these structures in the gap of magnetic flux concentrators (MFC) [6,7,8]. This strategy has been widely used for magnetic sensors [9,10,11], and has pushed the field detection level of SVs down to sub-nanoTesla range at room temperature [12,13].

In fact, discrete and integrated MFC solutions of very large dimensions (hundreds of μm to tens of mm), large gaps (>4 μm) [11,12,14,15,16] and tapered profiles [13] have delivered an increase in the field sensitivity in manifold when coupled to micrometric sensors. Drljaca *et al.* have reported gains in performance of 300 times using macroscopic discrete MFCs [11], being in contrast with gains of 3 to 6 times obtained for long integrated thin-fim MFCs (>500 × 500 μm${}^{2}$), with gaps of 10 to 56 μm [8,15,16]. Still, sensitivity gains for micrometric SV within ∼2400 × 900 μm${}^{2}$ MFCs and gaps of few μm are reported to be around 20 to 40 times [12,13].

The MFC’s material, geometry and length are well known to influence the gains in sensitivity [8,11], but the gap between MFCs poles is also a key parameter to control the field gain factor. Silva *et al.* showed systematically that smaller air gaps minimize magnetic flux leakage and provide a more effective concentration of the magnetic field upon the sensor with direct consequence of increased sensor sensitivity [8]. However, and since MFCs are usually composed of hundreds of nanometers thick films [7], defining poles with gaps from few μm down to sub-μm sizes is an important microfabrication challenge, which may lead to major improvements in field concentration gain factors.

**Figure 1:**
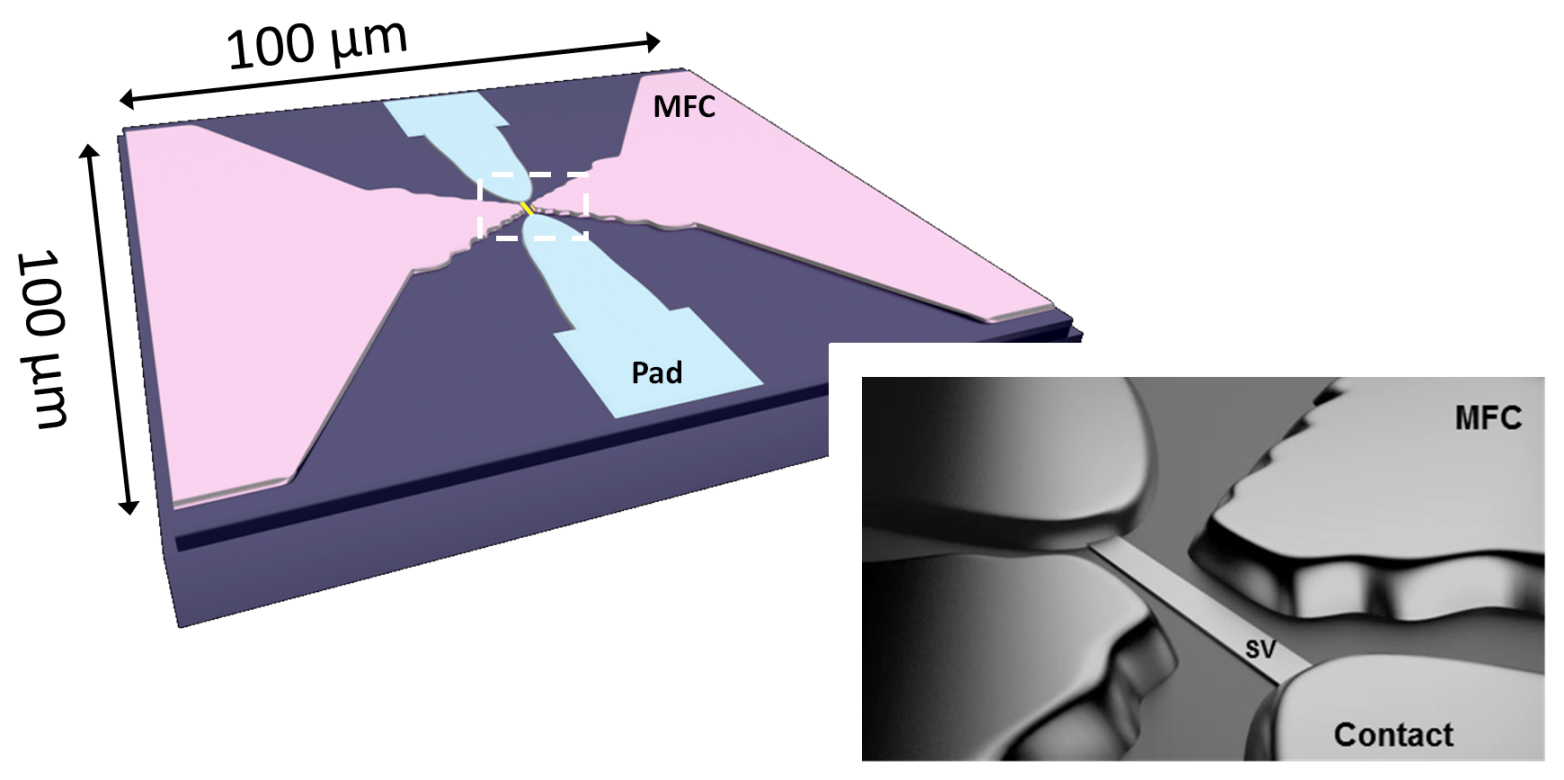
Illustrations of the final device layout including the spin-valve (SV) nanosensor, the electrical contacts and the magnetic flux concentrators (MFCs), all within 100 μm by 100 μm area.

In this work, we report an ultra compact design including nanometric SV sensors placed within ∼1000 down to 400 nm distance from the tips of 6000 Å-thick MFC elements (Figure 1). Our strategy consists on reducing the MFC dimensions so that a small device footprint (100 ×100 μm${}^{2}$) can be demonstrated, while successfully compensating the pronounced field reduction [12,13] (consequence of smaller dimensions) by reducing the pole to pole distance. Finally, in the absence of the SV element, the developed liftoff process for the patterned MFCs yields an extremely narrow gap of 380 nm with clearly defined boundaries.

## 2. Experimental Section

A bottom-pinned SV sensor with a synthetic antiferromagnetic (SAF) pinned-layer and synthetic-ferrimagnetic (SF) [17,18] free layer was deposited by ion beam in a Nordiko 3000 tool [19] (Nordiko Technical Services Ltd, Hampshire, UK) and consists of Si/Al${}_{2}$O${}_{3}$ 1000/Ta 20/Ni${}_{80}$Fe${}_{20}$ 30/Mn${}_{75}$Ir${}_{25}$ 80/Co${}_{80}$Fe${}_{20}$ 33/Ru 8/Co${}_{80}$Fe${}_{20}$ 33/Cu 25/Co${}_{80}$Fe${}_{20}$ 20/Ni${}_{80}$Fe${}_{20}$ 20/Ru 8/Ni${}_{80}$Fe${}_{20}$ 25/Ta 100 (thickness in Å). The samples were annealed at 250 ${}^{\circ }$C for 15 min to set the exchange bias at the pinned layer. The unpatterned sample exhibits an exchange coupling field of $H_{\mathrm{ex}}\approx 1840\;\mathrm{Oe}$, coercivity of $H_{c}\approx 0.5\;\mathrm{Oe}$ and an offset field $H_{o}\approx 33\;\mathrm{Oe}$. The SV stack was then patterned by electron beam lithography (EBL) using a 500 nm thick negative resist with a dose of 78 μC/cm${}^{2}$ for 20 kV, followed by an ion milling etching to define into a rectangular shape with nominal size of 13 × 0.5 μm${}^{2}$ (length (L) × height (*h*)), with triangular-shaped ends to decrease contact resistance [3]. After resist stripping, the structures yield an average *h* = 520 nm. The electrical contacts to the nanoSV were also defined by EBL and liftoff of Ru 400/AlSiCu 3000/TiWN${}_{2}$ 150 (thickness in Å), using PMMA as positive resist with a dose of 200 μC/cm${}^{2}$ for 10 kV.

**Figure 2:**
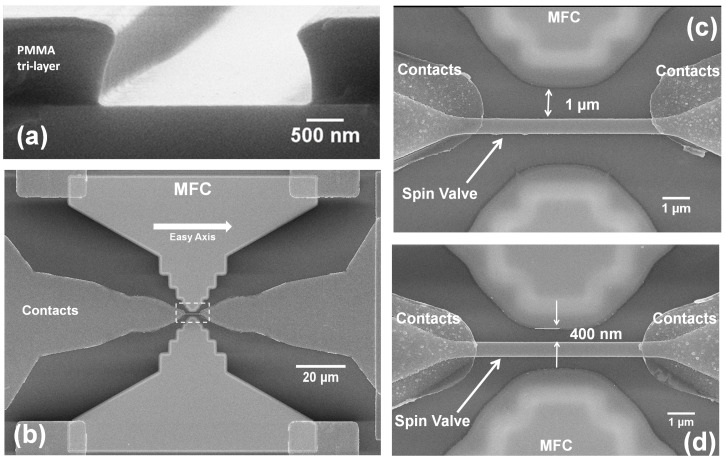
(**a**) Cross section SEM view of the tri-layer PMMA after electron beam lithography (EBL) exposure and development; (**b**) SEM top view of the full device and (**c**,**d**) detailed view of the largest and smallest gaps and sensor to pole distances fabricated using this strategy.

**Figure 3:**
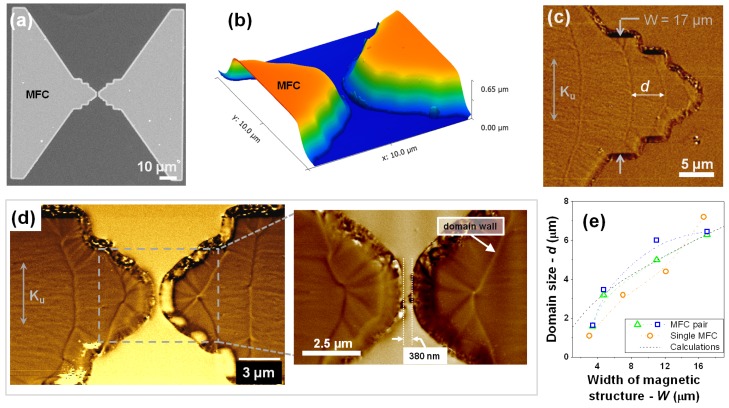
(**a**) SEM top view of a pair of patterned MFCs elements (6000Å-thick CZN) and (**b**) corresponding atomic force microscopy (AFM) topography view centered at the gap region, highlighting the clean liftoff profile without residues. Magnetic Force Microscopy (MFM) images of the poles domain structure at remanence in a (**c**) single and (**d**) pair of patterned MFCs. Inset: detail for the domain configuration of the smallest gap defines without SV sensor; (**e**) Comparison between calculated and measured domain size.

The patterned sensors exhibit a giant magnetoresistance (MR) of ∼5.0%, an overall resistance of ∼230 Ω (area between contacts × height = 4 × 0.5 μm${}^{2}$), yielding a sensitivity ∼0.02%/Oe. These sensors were then incorporated into the gap of two MFCs composed of an amorphous alloy of Co${}_{93.5}$Zr${}_{2.8}$Nb${}_{3.7}$ (CZN) 6000 Å-thick deposited by sputtering. CZN shows a saturation magnetization of 1100 $\mathrm{emu}/{\mathrm{cm}}^{3}$, easy axis $H_{c}$ = 1.7 Oe, hard-axis anisotropy field ($H_{k}$) of 13 Oe and a magnetic permeability ($\mu_{r}$) of 1200. The MFCs were patterned by EBL and liftoff using a 1.2 μm thick tri-layer PMMA positive resist (two layers of AR-P679 and one layer of AR-P649), coated at 1.5 krpm for 30 s followed by a 160 ${}^{\circ }$C baking for 4 min (for each layer). The EBL exposure was then divided into two steps, aiming at the optimum compromise between time and feature resolution: (i) a high definition procedure with a beam step size of 30 nm was used to define the MFCs pole; while (ii) a larger beam step size (>50 nm) was used to pattern the larger MFC features (away from pole). Figure 2a shows the slightly undercut profile obtained after development for 80 s in MIBK. Such process guarantees that an extremely narrow pole-sensor distance of 400 nm is achieved with 6000 Å-thick MFCs. In fact, we also demonstrate the fabrication of a pair of MFCs with the smallest patterned gap of 380 nm in the absence of a SV sensor and electrical contacts (inset of Figure 3d), thus setting the limit resolution for this procedure, which is highly dependent on the resist thickness. These are challenging goals to achieve at wafer level with patterned films defined by etching, since a safe end-point would require additional oxide protection over the SV element. The latter would increase the baseline level for the MFC with respect to the SV element, decreasing further the field concentration effect and requiring more tight restrictions for high accuracy alignment. Figure 2b shows a top view SEM image of the full device, comprising the sensing element in gap, a pair of MFCs and electrical contacts. The MFCs were designed with an entrance of 100 μm, pole width of 2.5 μm, total length of 50 μm and pole-sensor distances from 1 μm down to 400 nm (Figure 2c,d).

The MFCs were also characterized by atomic and magnetic force microscopies (AFM/MFM) using a Bruker Dimension Icon with a NT-MDT low moment magnetic tip enclosing a 15 nm CoCr coating. The tip resonance frequency was around 70 kHz, with a drive frequency of 65.85 kHz, amplitude of 1046 mV and a Q-factor of 162. The device transfer curves were measured with a dc two-point probe method within a field window of ±2 kOe at room temperature.

## 3. Results and Discussion

Figure 3a,b show the SEM view and corresponding AFM profile of a pair of MFCs after patterning. The MFCs exhibit a well defined gap with no liftoff residues and very clean boundaries with ∼65${}^{\circ }$ profile. Figure 3d highlights the limit sub-micrometric gap that we were able to achieve with our optimized process providing a resolved distance between poles. The MFCs poles were designed with rectangular shape, hence reinforcing the induced magnetic anisotropy during deposition with shape anisotropy. In this way, one promotes a magnetization orientation parallel to the SV longest direction minimizing magnetostatic coupling at zero field and ensuring a linear M(H) response. Although rounding of the MFC edges occurs, consequence of the EBL resolution for this thick resist layer, the concentrators still exhibit the expected domain structure (Figure 3c,d), with in-plane μm-size domains widths and remanent magnetization following the lateral edge profile. Figure 3e displays a consistent trend between the domain width measured from the MFM images and their theoretical size estimated for closure domain structures. For the latter, the domain width (*d*) can be approximated by $d∼{\left(2W\sqrt{\frac{A}{K_{u}}}\right)}^{\frac{1}{2}}$ [20,21], where *W* is the width of the magnetic structure, $A=1\times 10^{-6}$ erg/cm the exchange constant and $K_{u}=H_{k}M_{\mathrm{sat}}/2=7.1\times 10^{3}$ erg/cm${}^{3}$ the uniaxial anisotropy for CZN.

In this geometry, local canting of the magnetization consequence of the rounded edges and slope of the MFCs walls, added to the presence of local stray fields due to finite sizes features and defects, can originate preferential pining sites for domain walls [4,13,20,22], leading to deviations from the perfect arrangement of closure domain structures (Figure 3c) [20], as observed elsewhere [21]. The inset of Figure 3d shows a detail of the magnetic domain arrangement for the smallest gap distance achieved without sensor in gap. The difference in MFM contrast (brigth/dark) visible along the MFCs border and between the MFC tips suggests a magnetostatic pole-to-pole coupling at remanence. This behavior can lead to sensor coercivity, or decrease the efficiency of flux lines channeling through the SV magnetic material [21].

These results suggest the existence of a limit dimension for the smallest gap which ensures an efficient flux guiding through the sensor in-gap. For single-layer 6000 Å-thick CZN MFCs, a gap = 380 nm is bellow such limit size, where the shape and characteristics of the sensor’s output curve can be significantly affected. Still, pole-to-pole coupling can be mitigated by laminating the MFC materials [13,21,23], allowing one to push the gap distance to the process fabrication limits.

**Figure 4:**
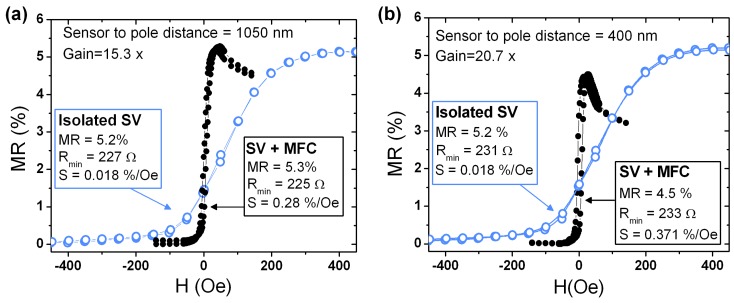
Transfer curves (I${}_{\mathrm{bias}}$ = 100 μA) for selected devices displaying the maximum sensitivity gain achieved, comparison the isolated SV curve with the corresponding device including the MFCs with a pole to sensor distance of (**a**) 1050 nm, the largest fabricated and (**b**) 400 nm, the smallest fabricated.

Figure 4 shows the transfer curves of selected devices with the largest pole to sensor distance of 1050 nm and the smallest of 400 nm. The initial curves of the isolated SV nanosensor is compared with the corresponding one when placed in the MFCs gap, displaying the expected increase in sensitivity upon MFC incorporation. Overall, a clear increase in the sensitivity gain with decrease in pole-sensor distance is visible, as a direct consequence of a more efficient field concentration in gap. A maximum gain of 15.3 was achieved for a pole-sensor distance of 1050 nm corresponding to a sensor sensitivity of 0.28%/Oe (Figure 4a). A maximum gain of 20.7 was obtained for the smallest pole-sensor distance of 400 nm, yielding a sensitivity of 0.37%/Oe, increasing from 0.018%/Oe achieved in the isolated SV (Figure 4b). Compared to Guedes *et al.* [12], we were able to decrease the full device footprint by ∼19 times while keeping a similar sensitivity gain (Gain = 20×). Furthermore, the offset field was also positively affected by the MFCs, from an average of ∼70 Oe in the isolated SV sensor decreasing to ∼4 Oe when MFCs are incorporated, according to the obtained gain. A top sensitivity of 0.46%/Oe was achieved for these devices for a pole-sensor distance of 550 nm. Finally, no significant dependence of coercivity with MFCs pole distance was observed in all measured structures.

**Figure 5:**
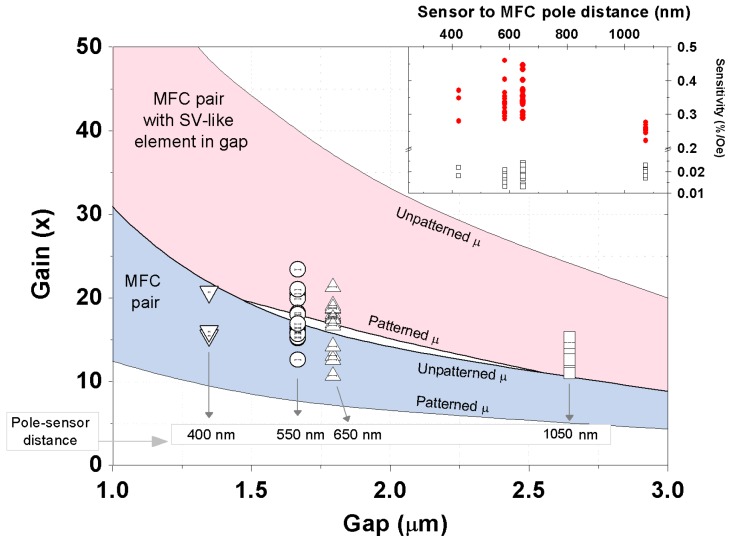
Experimental sensitivity gains obtained for all 47 measured devices, showing the dispersion of the results for each fabricated gap. Error bars in XX axis represent the deviations from the nominal gap size accessed by SEM. Full black line displays the trend obtained for the field gain by FEM simulations. Inset: Sensitivity values obtained for the isolated nanoSVs (black squares) and full devices (red dots) for all measured structures.

Figure 5 displays the field sensitivity gain measured for a total of 47 structures. The real gap size was characterized by SEM for several devices of each type with deviations of less than 2.2% from the nominal size. A slightly higher dispersion in the gains is observed for smaller gaps, consequence of more challenging MFC to sensor alignment precision. Figure 5 also shows the field amplification obtained from 3D finite element method simulations. The MFC geometry considered was based on the same mask layout for lithography with varying gaps from 1 up to 3 μm. The field gain is then obtained from the ratio between $H_{gap}/H_{external}$, where $H_{external}$ is the fixed field applied outside and $H_{gap}$ the one measured in gap. As input parameters we used the unpatterned CZN $\mu_{r}$, and the permeability corrected for patterned elements ($\mu_{p}$) [24], which according to Perrin *et al.* [25] yields $\mu_{p}$(CZN) ≈ 93. To further approximate simulations to the fabricated device, we have included a stripe of magnetic element in gap (height = 500 nm; $\mu_{p}$(stripe) ≈ 67) to mimic the SV nanosensor [25]. Our calculations yielded two distinct intervals of results, limited at top and bottom by the $\mu_{r}$ and $\mu_{p}$, respectively. As expected, one obtains an increasing gain with decreasing gaps as a consequence of lower losses in field concentration. A more efficient field concentration is achieved with the magnetic element in gap.

For larger gaps, the experimental points fall well within the simulations range considering the magnetic stripe in gap and $\mu_{p}$. As the gap size decreases, more dispersion is observed, with the data distributed among both predicted ranges. This suggests that when patterned closely, coupling or complex magnetization rearrangement near the MFC tips can appear (e.g., Figure 3d), affecting the guiding of the flux lines through the nanosensor. The latter is completely overlooked in this simple model. Nevertheless, our simulation shows a good agreement with experimental results, displaying feasibility for a robust estimation of the expected gain, particularly for gaps larger than 1.8 μm.

## 4. Conclusions

In conclusion, we have developed magnetic flux concentrators coupled to nanometric spin valve sensors with minimum pole-sensor distances of 400 nm. A decrease in the concentrators size was therefore compensated by decreasing the gap to improve flux guiding efficiency. The fabrication process requires three electron beam lithographies, one ion milling etching and two liftoff steps, achieving the smallest gap width of 380 nm for a 6000 Å-thick CZN MFC pair. The patterned MFCs exhibited a clean liftoff profile at the tip with closure domain structures at remanence. This process can also be extended to define robust and thick electrical contacts to current-in-plane nanosensors, leading to an extremely small active sensing area.

Maximum gains in sensitivity between 20× and 24× were obtained for pole-sensor distances below 600 nm and sensitivities up to 0.46%/Oe achieved for GMR sensor height of 500 nm. In addition, the incorporation of MFC has a positive impact on the large curve offset reducing it from 70 Oe to 4 Oe. Finally, arrays of these high performance hybrid devices with improved sensitivity levels and reduced footprint can be designed in a matrix configuration to perform high resolution magnetic field mapping with 100 × 100 μm${}^{2}$ pixel. Such design may require adjustments to the electrical leads layout to ensure a maximized spatial resolution, and to the MFC geometry to ensure efficient flux concentration and avoid magnetic coupling between neighboring pixels.
